# Prolonged fasting elicits increased hepatic triglyceride accumulation in rats born to dexamethasone-treated mothers

**DOI:** 10.1038/s41598-017-10642-1

**Published:** 2017-09-04

**Authors:** Lucas Carminatti Pantaleão, Gilson Murata, Caio Jordão Teixeira, Tanyara Baliani Payolla, Junia Carolina Santos-Silva, Daniella Esteves Duque-Guimaraes, Frhancielly S. Sodré, Camilo Lellis-Santos, Juliana Camargo Vieira, Dailson Nogueira de Souza, Patrícia Rodrigues Gomes, Sandra Campos Rodrigues, Gabriel Forato Anhe, Silvana Bordin

**Affiliations:** 1 Department of Physiology and Biophysics, Institute of Biomedical Sciences, University of Sao Paulo, Sao Paulo, Brazil; 2 Department of Pharmacology, Faculty of Medical Sciences, State University of Campinas, Campinas, Brazil; 3 Institute of Environmental, Chemical and Pharmaceutical Sciences, Federal University of Sao Paulo, Diadema, Brazil

## Abstract

We investigated the effect of dexamethasone during the last week of pregnancy on glucose and lipid metabolism in male offspring. Twelve-week old offspring were evaluated after fasting for 12-hours (physiological) and 60-hours (prolonged). Physiological fasting resulted in glucose intolerance, decreased glucose clearance after pyruvate load and increased PEPCK expression in rats born to dexamethasone-treated mothers (DEX). Prolonged fasting resulted in increased glucose tolerance and increased glucose clearance after pyruvate load in DEX. These modulations were accompanied by accumulation of hepatic triglycerides (TG). Sixty-hour fasted DEX also showed increased citrate synthase (CS) activity, ATP citrate lyase (ACLY) content, and pyruvate kinase 2 (*pkm2*), glucose transporter 1 (*slc2a1*) and lactate dehydrogenase-a (*ldha*) expressions. Hepatic AKT2 was increased in 60-hour fasted DEX, in parallel with reduced miRNAs targeting the *AKT2* gene. Altogether, we show that metabolic programming by prenatal dexamethasone is characterized by an unexpected hepatic TG accumulation during prolonged fasting. The underlying mechanism may depend on increased hepatic glycolytic flux due to increased *pkm2* expression and consequent conversion of pyruvate to non-esterified fatty acid synthesis due to increased CS activity and ACLY levels. Upregulation of AKT2 due to reduced miRNAs may serve as a permanent mechanism leading to increased *pkm2* expression.

## Introduction

Since the proposal of the thrifty phenotype theory by Hales and Barker^1^, a growing body of evidence has corroborated the hypothesis that intrauterine growth restriction (IUGR) is associated with an increased risk of type 2 diabetes (T2D) later in life^2^. Observational studies indicated that IUGR could potentially increase the long-term risk for T2D by impairing insulin sensitivity and the first phase of insulin secretion^3, 4^. Thus, the causal relationship between IUGR and programmed glucose intolerance has been extensively tested in several animal models. Rats born to mothers subjected to a 50% food restriction exhibit permanent disruption in pancreatic beta cell development and glucose intolerance later in life^5, 6^. In addition to the limited availability of nutrients to the conceptus, excessive corticosterone levels observed in food-restricted pregnant rats are believed to play a role in metabolic programming of the offspring^7^.

Experimental data that support the thrifty phenotype theory have also shown that rats born to mothers treated with dexamethasone (DEX) during the last week of pregnancy have impaired glucose tolerance later in life^8, 9^. This effect seems to be particularly relevant for the hepatic metabolic changes found in rats born to DEX-treated mothers, as these animals have increased levels of phosphoenolpyruvate carboxykinase (PEPCK), a limiting enzyme of gluconeogenesis^9^.

The role of the liver in energy metabolism, however, is not only attributed to its gluconeogenic capacity but also to an integral participation in lipid metabolism. The hepatic triacylglycerol (TG) content results from pools delivered to liver by chylomicron remnants or by non-esterified fatty acid (NEFA) uptake and esterification. In addition, hepatic sources of TGs are also derived from *de novo* NEFA synthesis from glucose^10^.

Insulin is the key endocrine signal that makes these metabolic pathways work dynamically during the fed-to-fasting transition. High insulin levels during the absorptive period act in the liver by suppressing gluconeogenesis and stimulating *de novo* NEFA synthesis^11^. Conversely, fasting conditions warrant high rates of hepatic gluconeogenesis and adipose tissue TG hydrolysis. Moreover, NEFA taken up by the liver will be either oxidized or esterified to TG, which will be secreted as very low-density lipoprotein (VLDL)^10^.

Evidence have shown that rats born to DEX-treated mothers are prone to accumulate high levels of TGs when exposed to high-caloric, fat-enriched diets during adult life^12, 13^. It is still uncertain if metabolic programming by prenatal DEX exposure is also characterized by an impaired adaptation of hepatic metabolism to prolonged nutrient deprivation. To address this issue, we evaluated the effects of long lasting fasting periods on multiple aspects of glucose and lipid metabolism in offspring born to DEX-treated mothers.

## Results

### Rats born to DEX-treated mothers exhibit glucose intolerance when subjected to overnight fasting

We initially assessed body weights at birth to verify that our experimental protocol replicated a well-known result of excess glucocorticoid in offspring^8^. As expected, the body weight at birth was reduced in those born to DEX-treated mothers. Control rats weighted 7.1 ± 0.2 g (n = 20), and DEX-offspring weighed 4.4 ± 0.1 g (n = 11) at birth (38% lower than control [CTL]; P < 0.05).

**Figure 1:**
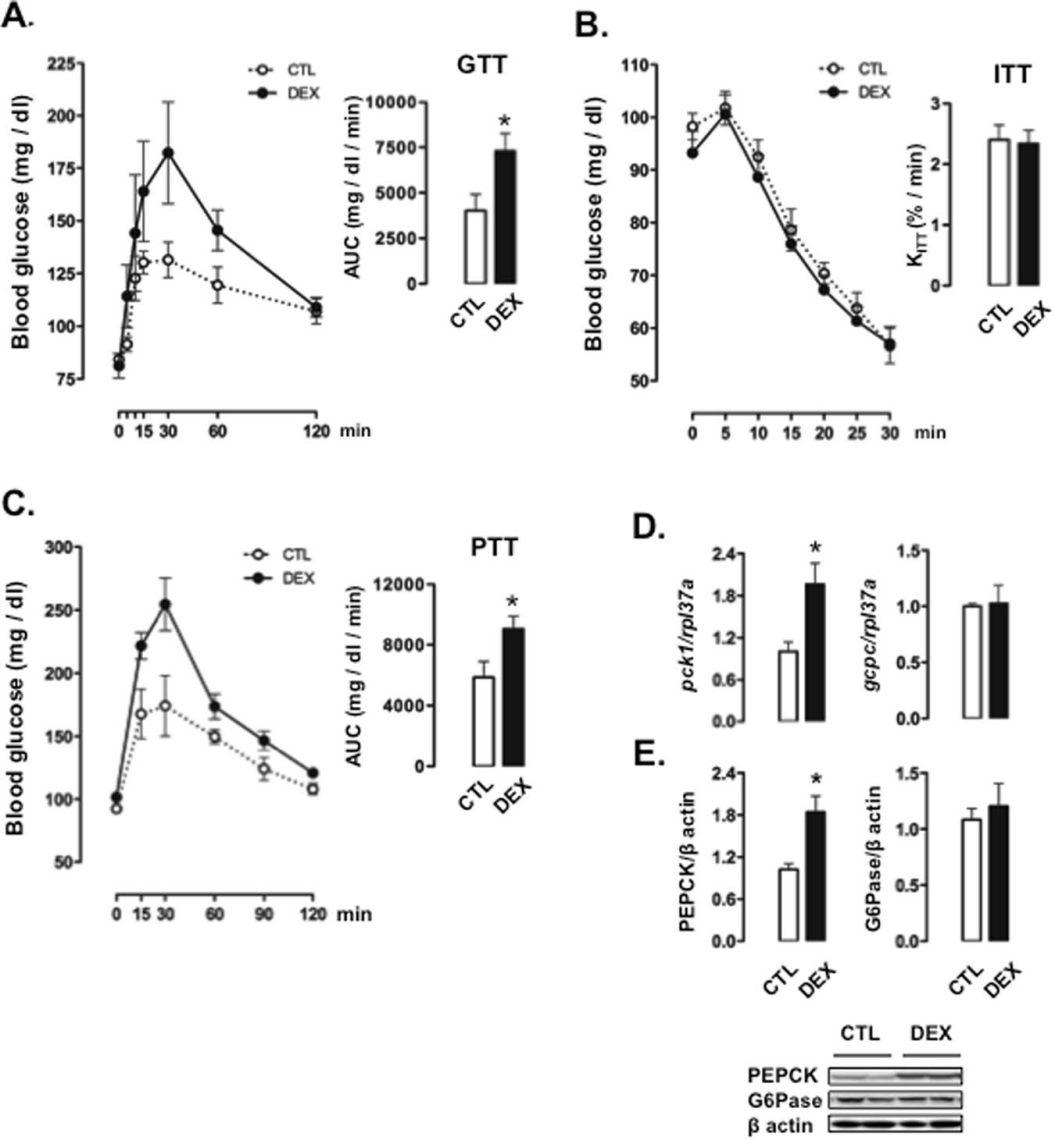
Glucose metabolism in 12-hour fasted rats born to DEX-treated mothers. (**A**) GTT, (**B**) ITT and (**C**) PTT were performed in 12-hour fasted CTL (rats born to untreated mothers) and DEX (rats born to DEX-treated mothers) rats. The AUCs were calculated for glucose tests and PTTs. The K_ITT_ was calculated for ITTs. (**D**) Hepatic determination of *pck1* and *gcpc* mRNA was normalized to *rpl37a* expression. (**E**) Hepatic determination of PEPCK and G6Pase was normalized to β actin. Data are shown as the mean ± S.E.M.; *p < 0.05 vs. CTL.

The metabolic programming in rats born to DEX-treated mothers was initially assessed in adult rodents subjected to a 12-hour fast. Changes in glycemia after a glucose load are depicted in Fig. 1A. Our data demonstrate that offspring born to DEX-treated mother exhibit glucose intolerance over the course of a glucose tolerance test (GTT), as shown by increased values of the area under the curve (AUC) obtained from the GTT (82% higher than those from CTL; P < 0.05).

Insulin tolerance tests (ITTs) were performed to assess putative changes in whole-body insulin sensitivity. The glucose decay after the load with exogenous insulin and the constant rate for glucose disappearance (K_ITT_) were similar between CTL and rats born to DEX-treated mothers (Fig. 1B).

To evaluate if the impaired glucose tolerance of rats born to DEX-treated mothers was associated with increased whole-body gluconeogenesis, we performed the pyruvate tolerance test (PTT). The glucose levels after a pyruvate load are shown in Fig. 1C. Our data reveal that rats born to DEX-treated mothers exhibited a reduced clearance of glucose after a challenge with pyruvate, as shown by a greater AUC observed in rats born to DEX-treated mothers (54% higher than that of CTL animals; P < 0.05).

Reduced clearance of glucose after a pyruvate load in rats born to DEX-treated mothers matched with an increased *pck1* expression in the liver (96% higher than CTL; P < 0.05). No differences were found in *g6pc* expression (Fig. 1D) or glucagon and insulin levels (not shown). Western blot analysis revealed that increased protein levels of PEPCK were present in the liver of rats born to DEX-treated mothers (80% higher than CTL; P < 0.05). Representative of *g6pc* gene expression, rats born to DEX-treated mothers showed unaltered levels of G6Pase protein (Fig. 1E).

### Rats born to DEX-treated mothers have increased tolerance to exogenous glucose when subjected to a prolonged fast

**Figure 2:**
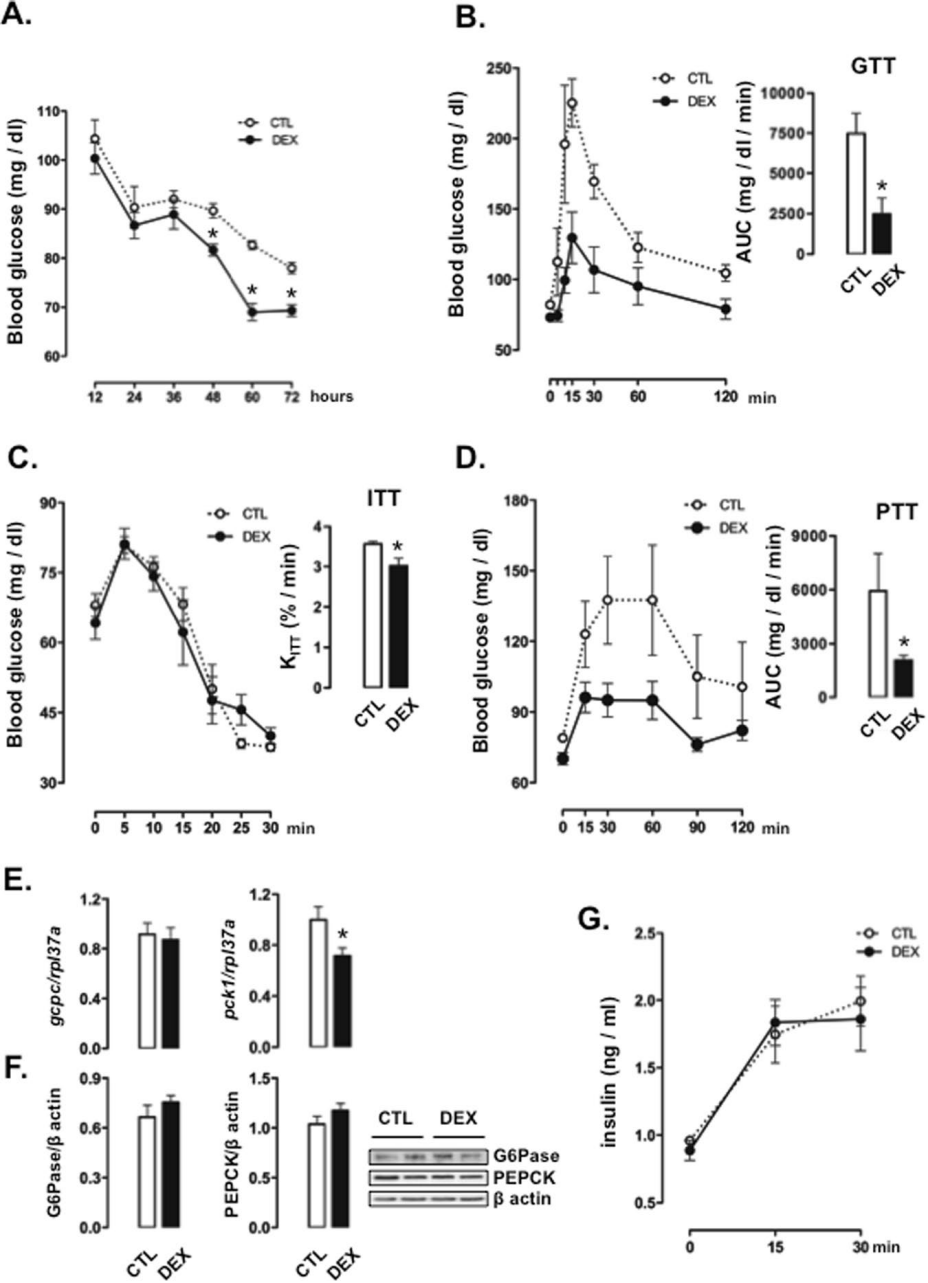
Glucose metabolism in 60-hour fasted rats born to DEX-treated mothers. (**A**) Blood glucose levels were determined at different time points in CTL (rats born to untreated mothers) and DEX (rats born to DEX-treated mothers) rats during a 72-hour fasting period. (**B**) GTT, (**C**) ITT and (**D**) PTT were performed in 60-hour fasted CTL and DEX rats. The AUCs were calculated for glucose tests and PTTs. The K_ITT_ was calculated for ITTs. (**E**) Hepatic determination of *pck1* and *gcpc* mRNA was normalized to *rpl37a* expression (**F**) Hepatic determination of PEPCK and G6Pase was normalized to β actin. (**G**) Circulating insulin was assessed at specific time points during the GTT. Data are shown as the mean ± S.E.M.; *p < 0.05 vs. CTL.

The initial analysis revealed that excess glucocorticoid during pregnancy resulted in glucose intolerance along with increased hepatic PEPCK expression and gluconeogenesis. To evaluate if these effects are sustained during a prolonged fast, we initially assessed fasting glycemia during progressive intervals of food deprivation. Surprisingly, we found that rats born to DEX-treated mothers exhibited lower glucose levels with prolonged fasting. Rats born to DEX-treated mothers showed reduced glucose level after a 48-, 60- and 72-hour fast (respectively, glucose levels were 13%, 18% and 13% lower than CTL; P < 0.05; Fig. 2A).

In an attempt to better characterize the metabolic response to a prolonged food deprivation period in rats born to DEX-treated mothers, we performed *in vivo* metabolic assays after a 60-hour fast. When subjected to a 60-hour fast, rats born to DEX-treated mothers presented lower glucose levels throughout the course of the GTT, which resulted in reduced AUCs (67% lower than CTL values; P < 0.05; Fig. 2B). Although rats born to DEX-treated mothers became more tolerant to glucose after the 60-hour fast, they exhibited discrete whole-body insulin resistance, as shown by reduced values of K_ITT_ (15% lower than CTL; P < 0.05; Fig. 2C).

In parallel to an increased tolerance to exogenous glucose, a 60-hour fast in rats born to DEX-treated mothers also resulted in lower glucose levels after a challenge with pyruvate. Thus, the AUCs obtained from the PTT in rats born to DEX-treated mothers were reduced when compared to those obtained from CTL rats (71% lower; P < 0.05; Fig. 2D). Livers obtained from 60-hour fasted rats born to DEX-treated mothers exhibited reduced levels of *pck1* (28% lower than CTL; P < 0.05) but unchanged levels of *g6pc* (Fig. 2E). This change in mRNA expression did not translate into changes in protein content because both PEPCK and G6Pase levels were unaltered in rats born to DEX-treated mothers (Fig. 2F).

Sixty-hour fasted CTL rats and 60-hour fasted rats born to DEX-treated mothers showed a similar increase in insulin levels in response to glucose. Both groups demonstrated a 90% increase in insulin levels at 15 min after the glucose injection. The values of circulating insulin remained similar between the two groups up to 30 min after the glucose load (Fig. 2G).

### Rats born to DEX-treated mothers have increased hepatic TG accumulation when subjected to a 60-hour fast

**Figure 3:**
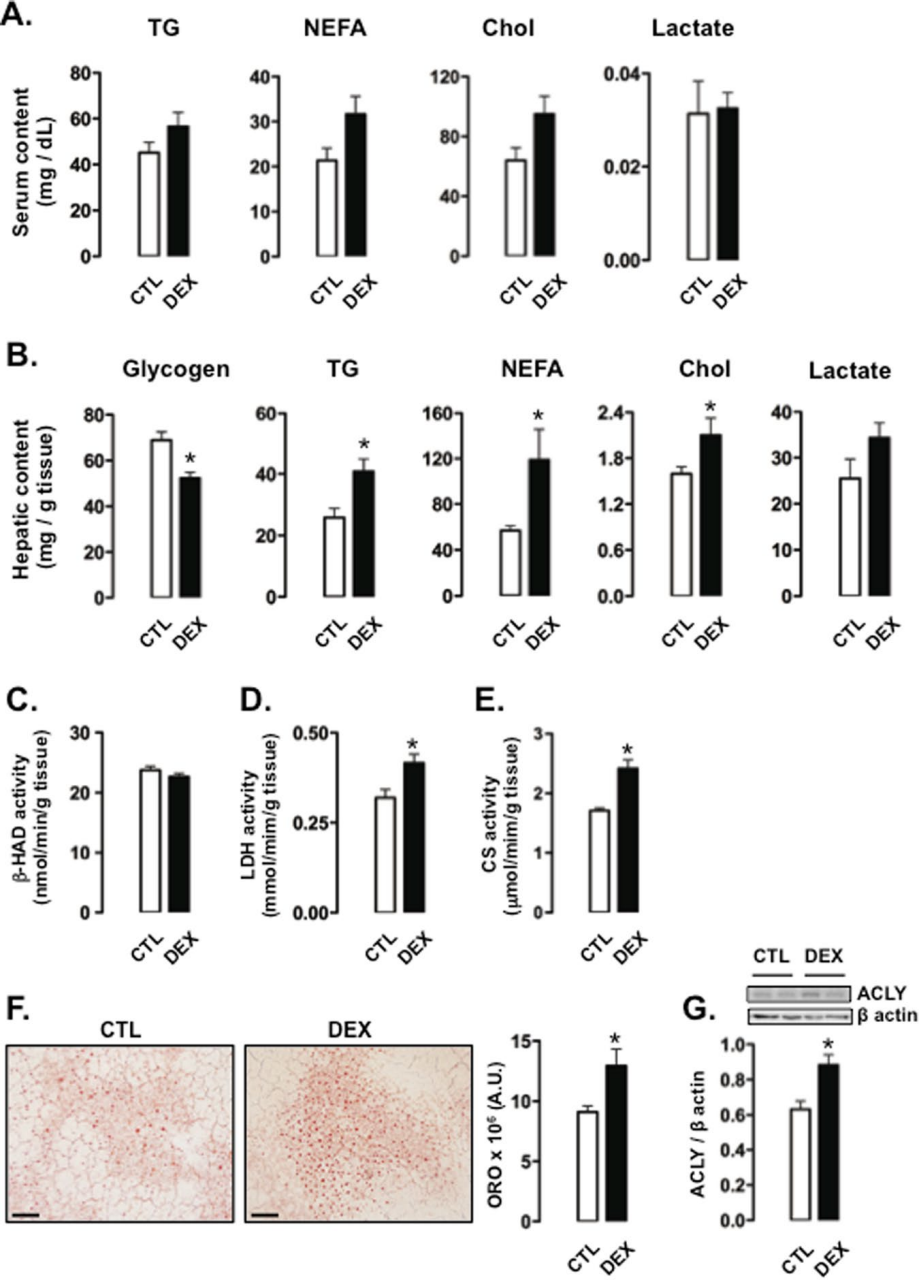
Parameters related to glucose and lipid metabolism in 60-hour fasted rats born to DEX-treated mothers. (**A**) Serum levels of TGs, NEFA, cholesterol (Chol) and lactate were determined in CTL (rats born to untreated mothers) and DEX (rats born to DEX-treated mothers) rats subjected to a 60-hour fast. (**B**) One set of liver samples was used to measure glycogen, TGs, NEFA, Chol and lactate. (**C, D** and **E**) A second set of liver samples was processed for the determination of enzymatic activity of (**C**) β-HAD, (**D**) LDH and (**E**) CS. (**F**) TG content was also assessed by staining liver sections with Oil Red O. (**G**) Hepatic determination of ACLY was normalized to β actin. Data are shown as the mean ± S.E.M.; *p < 0.05 vs. CTL.

To elucidate the fate of glucose and pyruvate after a prolonged period of food deprivation in rats born to DEX-treated mothers, we assessed circulating and hepatic parameters of lipid metabolism after subjecting the rats to a 60-hour fast. Circulating TGs, NEFA, cholesterol and lactate were similar between CTL rats and rats born to DEX-treated mothers (Fig. 3A). Hepatic levels of TGs, NEFA and cholesterol, however, were increased in rats born to DEX-treated mothers (59%, 110% and 32% higher than CTL, respectively; P < 0.05), and glycogen was reduced to 76% of CTL values (P < 0.05). The levels of hepatic lactate were similar between the two groups (Fig. 3B). Increased TG content in the liver of rats born to DEX-treated mothers was also detected by analysis of liver sections stained with Oil Red O (42% higher than CTL; P < 0.05; Fig. 3F). The activity of L-3-hydroxyacyl CoA dehydrogenase (β-HAD) was assessed as a representative step of fatty acid β-oxidation. Similar levels of β-HAD activity were found in the livers of CTL and rats born to DEX-treated mothers (Fig. 3C). Lactate dehydrogenase (LDH) and citrate synthase (CS) activities were increased in the liver of rats born to DEX-treated mothers (respectively, 30% and 41% higher than CTL; P < 0.05; Fig. 3D and E). The content of ATP citrate lyase (ACLY) was also increased in the liver of rats born to DEX-treated mothers (40% higher than CTL; P < 0.05; Fig. 3G).

**Figure 4:**
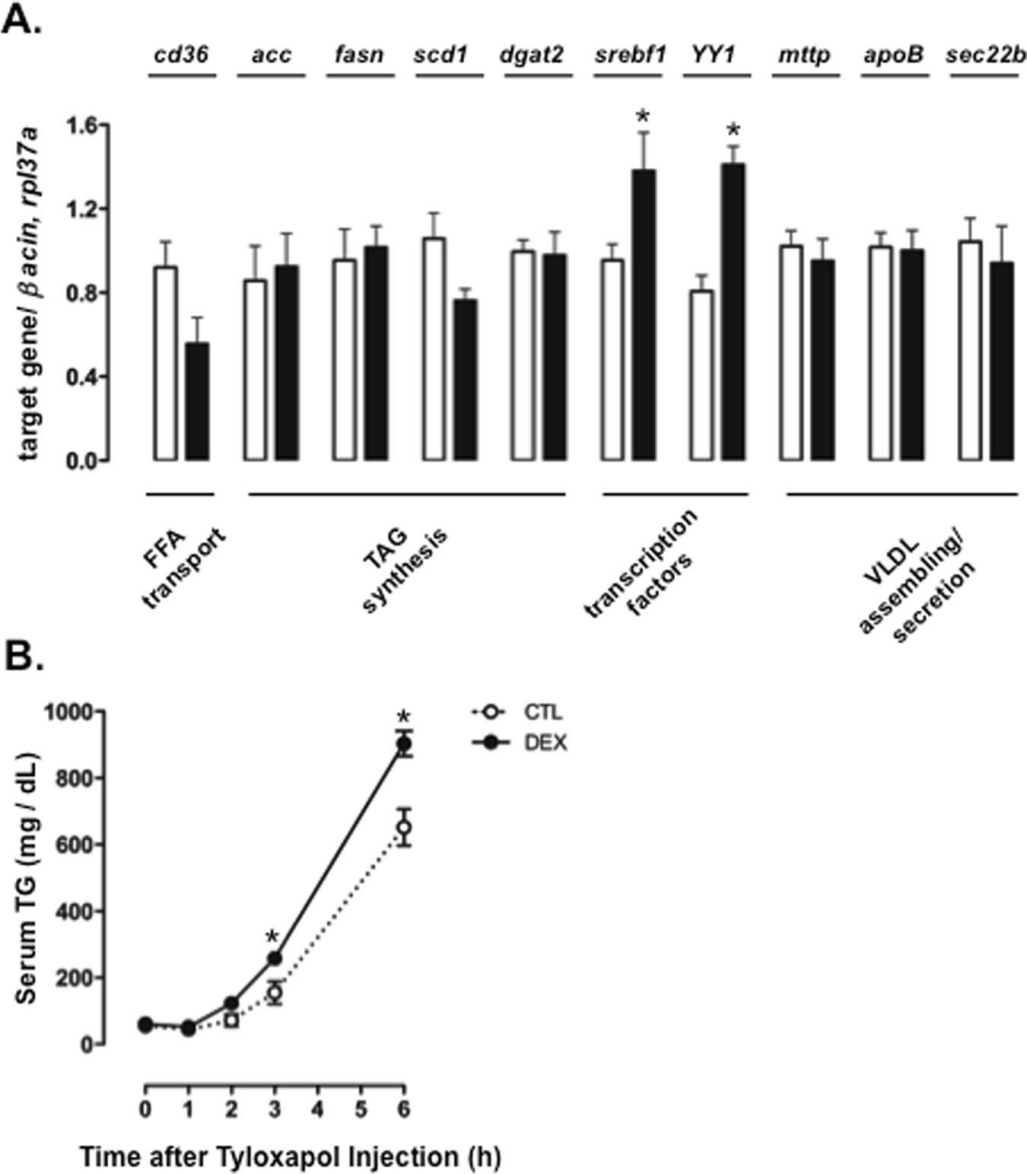
Expression of genes related to lipid metabolism and VLDL production in 60-hour fasted rats born to DEX-treated mothers. (**A**) Liver samples of CTL (rats born to untreated mothers) and DEX (rats born to DEX-treated mothers) rats subjected to a 60-hour fast were processed for determination of *cd36*, *acc*, *fasn*, *scd*, *dgat2*, *srebf1*, *YY1*, *mttp*, *apoB* and *sec. 22* mRNA. Data were normalized to *rpl37a*. (**B**) CTL and DEX rats fasted for 60 hours were subjected to tyloxapol injection. The serum TG levels were measured at different time points after injection. Data are shown as the mean ± S.E.M.; *p < 0.05 vs. CTL.

To better understand the increased levels of TGs in the liver of 60-hour fasted rats born to DEX-treated mothers, we analyzed the expression of several genes that encode proteins related to lipid metabolism. Two important transcription factors that control the expression of several enzymes related to *de novo* fatty acid synthesis and esterification into TGs, *srebf1* and *Yin Yang 1* (*YY1*), were evaluated. Both *srebf1* and *YY1* expressions were increased in the liver of rats born to DEX-treated mothers (respectively, 45% and 75% higher than CTL; P < 0.05). Expression of the enzymes related to *de novo* fatty acid synthesis acetyl CoA carboxylase (*acc*), fatty acid synthase (*fasn*) and stearoyl-CoA desaturase (*scd*), the fatty acid transporter CD36 and diacylglycerol acyltransferase 2 (*dgat2*) were similar between the groups. Similarly, we did not detect differences in the expression of proteins related to VLDL synthesis, assembly or secretion, such as apolipoprotein B (ApoB), microsomal transfer protein (MTTP) and SEC. 22b (Fig. 4A).

Hepatic secretion of VLDL-TG was also evaluated by assessing TG accumulation in blood after a challenge with tyloxapol, an inhibitor of lipoprotein lipase (LPL). Rats born to DEX-treated mothers showed a more pronounced capacity to accumulate TGs in the blood than the CTL rats when subjected to a 60-hour fast, as shown by increased TG concentrations at 3 and 6 hours after tyloxapol injection in rats born to DEX-treated mothers compared to CTL rats (39% and 66% higher than CTL, respectively; P < 0.05; Fig. 4B).

### Rats born to DEX-treated mothers have increased AKT2 levels and reduced putative miRNAs targeting AKT2 after a prolonged fast

**Figure 5:**
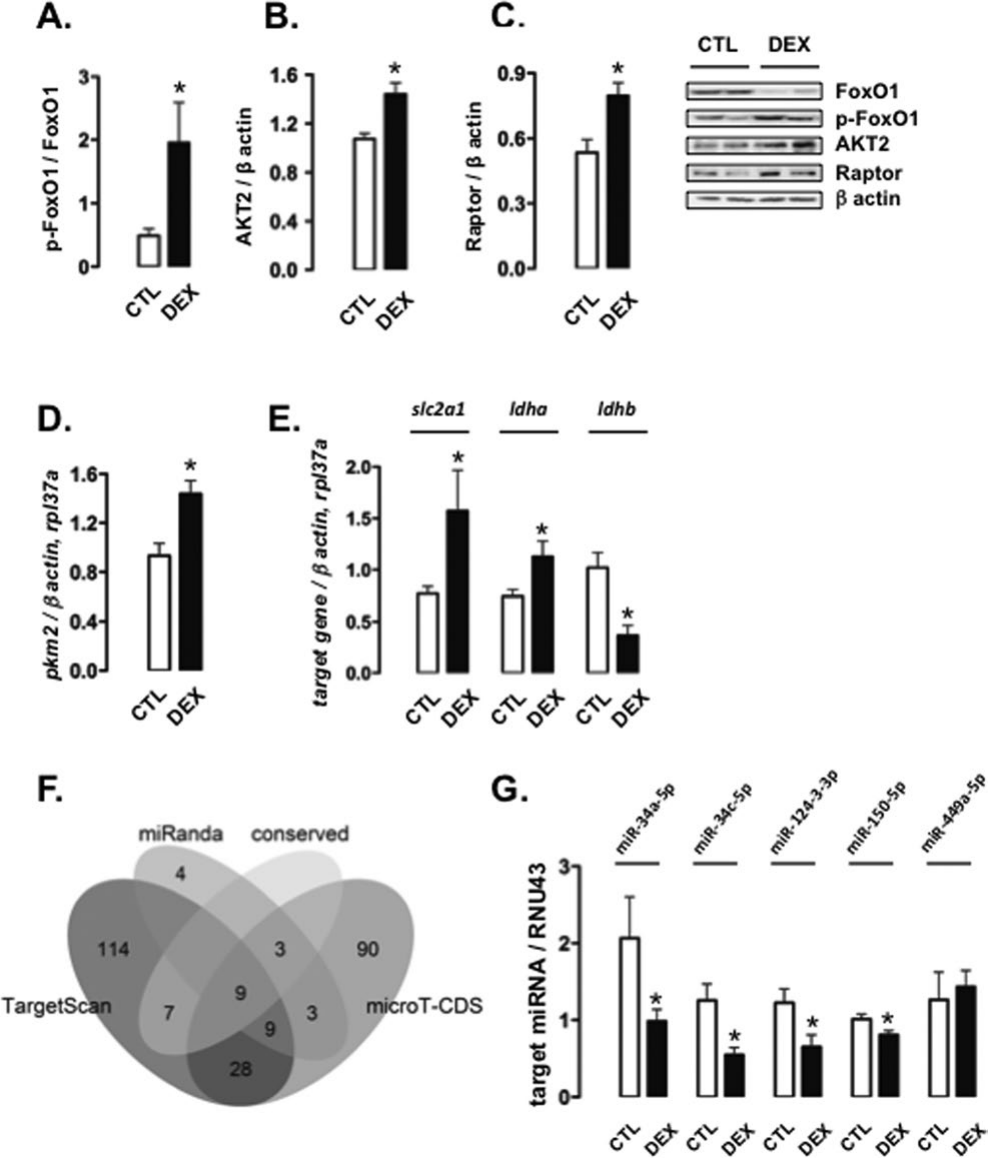
Signaling proteins and miRNAs in the liver of 60-hour fasted rats born to DEX-treated mothers. (**A**–**C**) One set of liver samples of CTL (rats born to untreated mothers) and DEX (rats born to DEX-treated mothers) rats subjected to a 60-hour fast was processed for western blot detection of (**A**) pFoxO1 normalized to FoxO1 and (**B**) AKT2 and (**C**) Raptor normalized to β-actin. (**D** and **E**) Liver samples of CTL and DEX rats fasted for 60 hours was processed for the determination of (**D**) *pkm2* and (**E**) *slc2a1*, *ldha* and *ldhb* mRNA normalized to *rpl37a* expression. (**F**) *In silico* analysis using 3 available algorithms (TargetScan, miRanda and microT-CDS) was performed to identify miRNAs likely to physically bind to *akt2* mRNA to control its expression. (**G**) Liver samples were also used for miR-34a-5p, miR-34c-5p, miR-124-3-3p, miR-150-5p and miR-449a determination, and the expression of each is normalized to RNU43 expression. Data are shown as the mean ± S.E.M.; *p < 0.05 vs. CTL.

We also evaluated the changes in insulin signaling that could have mediated the hepatic lipogenesis after a 60-hour fast. Rats born to DEX-treated mothers had increased forkhead box protein O1 (FoxO1) phosphorylation compared to CTL rats (299% higher than CTL; P < 0.05; Fig. 5A). AKT2 and Raptor protein content was also increased in the liver of rats born to DEX-treated mothers (respectively, 34% and 49% higher than CTL; P < 0.05; Fig. 5B and C). The expression of pyruvate kinase 2 (*pkm2*), known to be regulated by AKT2 and mammalian target of rapamycin (mTOR)^14, 15^, was also increased in the liver of 60-hour fasted rats born to DEX-treated mothers (54% higher than CTL; P < 0.05; Fig. 5D).

PKM2 modulates the expression of several genes, including *slc2a1* and lactate dehydrogenase a (*ldha*)^16^. Favoring the proposition that PKM2 is likely to play a functional role in our model, 60-hour fasted rats born to DEX-treated mothers showed an increased expression of *slc2a1* and *ldha* (respectively, 104% and 52% higher than CTL; P < 0.05). On the other hand, the expression of lactate dehydrogenase b (*ldhb)*, a marker of oxidative phosphorylation, was reduced in 60-hour fasted rats born to DEX-treated mothers (36% lower than CTL; P < 0.05; Fig. 5E).

Next, we investigated whether programmed miRNAs were involved in the regulation of hepatic AKT2 expression due to early DEX exposure. Although hundreds of transcripts were predicted by the algorithms, only 18 were commonly indicated by all databases (Fig. 5F). Among these, 9 displayed binding sites classified as highly conserved among species either by TargetScan or miRanda. We then analyzed the expression of the top 5 miRNAs with conserved sites in the *Akt2* mRNA 3’ untranslated region (3′UTR), according to the scoring system calculated by TargetScan (Supplementary Table S1).

Expression of miR-34a-5p, miR-34c-5p, miR-124-3p, and miR-150-5p (respectively, 52%, 56%, 47% and 20% lower than CTL; P < 0.05) but not miR-449a was reduced in the liver of 60-hour fasted rats born to DEX-treated mothers (Fig. 5G).

## Discussion

The present study presents evidence to show that metabolic programming induced by prenatal exposure to DEX is characterized by changes in glucose handling that depends on fasting duration. A physiological short-term fast elicits glucose intolerance hallmarked by increased pyruvate conversion into glucose and increased hepatic PEPCK in rats born to DEX-treated mothers. These changes are similar to previous findings^9^. In contrast, rats born to DEX-treated mothers exhibited increased tolerance to exogenous glucose compared to CTL rats after a prolonged period of food deprivation.

Interestingly, this adaptation was not accompanied by increased insulin sensitivity; instead, there was a discrete insulin resistance. Thus, increased glucose uptake by insulin-sensitive tissues, such as skeletal muscle and white adipose tissue fat depots, are unlikely to account for the increased tolerance to exogenous glucose after a prolonged fast. We have also shown that the increase in insulin levels in 60-hour fasted rats born to DEX-treated mothers during the course of the GTT was similar to that of 60-hour fasted CTL rats. Thus, our data also rule out increased insulin secretion as the expected cause for increased glucose tolerance in 60-hour fasted rats born to DEX-treated mothers.

Metabolic programming induced by prenatal exposure to DEX is characterized by unaltered hepatic PEPCK content after a 60-hour fast. However, rats born to DEX-treated mothers, when fasted for 60 hours, showed a reduced conversion of exogenous pyruvate into glucose. Thus, the combined results of the GTT and the PTT suggest that adult rats exposed to DEX during fetal life, under the condition of prolonged food deprivation, are likely to display increased hepatic consumption of glucose and glycolysis intermediates, such as pyruvate, to generate high amounts of Kreb cycle intermediates.

The increase in *slc2a1* and *pkm2* expression in the liver of 60-hour fasted rats born to DEX-treated mothers supports the notion of increased glycolysis rate. The increase in CS activity, in turn, supports the notion that the bulk of pyruvate (either exogenous or derived from glucose), rather than serving as a gluconeogenic substrate, is being converted into citrate in the liver of 60-hour fasted rats born to DEX-treated mothers.

Our data also support the proposition that citrate is probably being directed to *de novo* synthesis of NEFAs, given that 60-hour fasted rats born to DEX-treated mothers also had increased hepatic levels of ACLY, TGs and NEFAs.

Our study further suggests that increased hepatic *de novo* synthesis of NEFA is responsible for the hepatic TG accumulation in 60-hour fasted rats born to DEX-treated mothers. First, these rats showed no differences in hepatic expression of CD36, a transporter of circulating NEFA^17^. Second, circulating NEFA after a 60-hour fast was not altered by prenatal exposure to DEX. Thus, it is unlikely that increased hepatic NEFA uptake from the blood stream and its consecutive esterification would account for the differences in hepatic TG levels. Additionally, no evidence for a reduction in hepatic fatty acid β-oxidation has been found, as the rats born to DEX-treated mothers did not exhibit altered activity of hepatic β-hydroxyacyl-CoA dehydrogenase (β-HAD) after a 60-hour fast.

Our data have also shown that hepatic TG accumulation in the liver of rats born to DEX-treated mothers is equally unlikely to result from impaired VLDL production. This conclusion was drawn from the results of serum TG concentration after tyloxapol injection. Sixty-hour fasted rats born to DEX-treated mothers exhibited a more pronounced increased in serum TG concentration after tyloxapol injection when compared to 60-hour fasted CTL rats. Importantly, no changes were observed in the expression of *mttp*, *apoB* or *sec. 22b*, key players in hepatic VLDL production, assembly and secretion^18, 19^.

We believe that the higher levels of serum TG concentration found in rats born to DEX-treated mothers after tyloxapol injection are probably due to higher TG content in VLDL, rather than an increased in the number of VLDL particles. The increased TG content within VLDLs might be a direct consequence of increased hepatic TGs observed in these rats. In accordance with this hypothesis, it was already demonstrated that patients with nonalcoholic fatty liver disease (NAFLD) have increased serum concentration of TGs but not ApoB. Thus, the association between serum TGs and hepatic steatosis is suggested to be result of higher TG enrichment of VLDL particles^20^.

Additionally, increased serum TG concentration in 60-hour fasted rats born to DEX-treated mothers only becomes evident after an injection of tyloxapol, an LPL inhibitor. Thus, it is possible that, under prolonged fasting conditions, increased LPL-mediated TG depuration from VLDL in rats born to DEX-treated mothers yields unchanged serum TG levels. Another aspect that warrants consideration is that greater VLDL levels with a higher TG content were already described to have a lower half-life, being quickly removed from the blood stream^21^.

Experiments with liver-specific knockout mice have demonstrated that hepatic AKT2 is required for *de novo* lipogenesis and TG accumulation^22^. Downregulation of *de novo* lipogenesis in mice lacking hepatic AKT2 was shown to correlate with concomitant reduction in the expression of *srebf1* (the gene that encodes SREBP-1c) and its target genes, such as *fasn*, *scd* and *acc* ^22^. In fact, data collected show that SREBP-1c activation plays an important role in insulin-induced *de novo* lipogenesis through AKT2^23^. Furthermore, mTORC1 was described to be a relevant step downstream of AKT that leads to SREBP1c activation and *de novo* lipogenesis^24, 25^. Although we have found that a 60-hour fast increased expression of *srebf1*, AKT2 and Raptor (a specific component of the mTORC1 complex;^26^) in the liver of rats born to DEX-treated mothers, we have found no concomitant increase in the expression of enzymes that mediate NEFA synthesis, such as *acc*, *fasn* and *scd*. Thus, it is quite possible that *Srebp1* is not playing a role in increased hepatic TG accumulation in rats born to DEX-treated mothers. This is plausible because *Srebp1* is described to require other co-activators, such as NF-Y/CBF, CREB/ATF and Sp1 to efficiently stimulate gene expression^27, 28^.

Therefore, we propose that increased lipogenesis observed in our model results from increased metabolic flux through glycolysis with compensatory mitochondrial citrate synthesis. Citrate, rather than entering the Kreb cycle, is preferentially serving as substrate for ACLY to promote fatty acid synthesis. This metabolic adaptation, widely described in cancer cells and known as the Warburg effect or aerobic glycolysis, is hallmarked by high glycolytic rates, even in the presence of oxygen^29^. Metabolic intermediates directed toward *de novo* lipogenesis are important to the Warburg effect that enables cancer cell growth^30^.

The cause of the Warburg effect can rely on both mitochondrial defects and enhanced glycolytic activity^31^. Notably, AKT2 was described as one mediator of the growth of liver cancer cells by stimulating the expression of PKM2^15^. AKT2-mediated control of PKM2 is also relevant for liver fat accumulation^32^. PKM2 expression and the Warburg effect were also described to depend on the mTOR complex^14^. This regulation seems to be bidirectional, as PKM2 can also activate the mTOR complex 1 (mTORC1) in cancer cells^33^. Aside from the established role of PKM2 in catalyzing pyruvate synthesis, this protein also functions as a transcriptional co-activator^34^. Upon stimulation, PKM2 translocates into the nucleus and stimulates the expression of *slc2a1* (GLUT1) and LDHA expression by a c-myc-dependent mechanism^16^.

Collectively, our data support the unprecedented notion that metabolic programming by prenatal DEX is hallmarked by a Warburg-like effect, which becomes apparent during a prolonged fast. The livers of rats born to DEX-treated mothers displayed increased AKT2 and Raptor protein expression and *pkm2* gene expression. Genes downstream to PKM2, such as *slc2a1* and LDHA, were also increased. Alternatively, reduced levels of LDHB further indicates lower rates of oxidative phosphorylation. Increased FoxO1 phosphorylation strengthens the hypothesis that a 60-hour fast increases AKT activity in the liver of rats born to DEX-treated mothers. The reduction in FoxO1 content is likely to result from its degradation. AKT-mediated FoxO1 phosphorylation mediates its translocation from the nucleus to the cytoplasm, where FoxO1 undergoes ubiquitination and proteasome degradation^35^. A plausible explanation for the increase in Raptor expression relies on a putative inhibition of its proteasome-mediated degradation due to increased AKT2 levels. Accordingly, proteasome-mediated degradation of Raptor is induced by pharmacological AKT inhibition^36^.

With an attempt to understand the epigenetic basis of the programmed increased in AKT2 levels, we have searched for potential miRNAs that regulate AKT2 expression. *In silico* analysis scored nine miRNAs as commonly conserved putative regulators of the rat AKT2 gene. Four of these miRNAs were downregulated in the liver of rats born to DEX-treated mothers (miR-34a-5p, miR-34c-5p, miR-124-3-3p and miR-150-5p). Importantly, human *AKT2* was previously described as a validated target for miR-124-3p^37^, and its binding sequence in both human and rat mRNAs is preserved and shares complete homology. Conservation is an important feature when predicting miRNA target sequences, as true binding sites are likely to be preserved under selective pressure^38^. Additionally, an increase in phosphorylated AKT after the inhibition of the miR-34 family has been observed^39^. Thus, our data indicate that AKT2 is likely to be regulated by miRNAs in the liver of rats born to DEX-treated mothers.

In summary, the present data describe an unknown feature of metabolic programming induced by prenatal DEX that is characterized by improper *de novo* lipogenesis of the liver during a prolonged fast. This metabolic adaptation leads to hepatic TG accumulation and is likely to result from increased glycolytic flux as a consequence of upregulated hepatic *pkm2*. Increased *pkm2* expression is paralleled by increased protein expression of AKT2 and Raptor. We also believe that differential miRNA expression serves as a putative mechanism underlying the increase in hepatic AKT2 content.

## Methods

### Experimental design and animal treatment

Nulliparous Wistar rats (8 weeks old) were treated with DEX (0.2 mg/kg/day) diluted in the drinking water from the 14^th^ to the 19^th^ day of pregnancy, as previously described (Gomes *et al*., 2014). The offspring were weaned on the 21^st^ day of life and maintained on standard chow and water *ad libitum*. At 12 weeks old, the offspring were fasted for 12 h or 60 h prior to the experiments.

All studies were performed according to the guidelines of the Brazilian College for Animal Experimentation (COBEA) and were approved by the Ethics Committee on Animal Use at the Institute of Biomedical Sciences, University of Sao Paulo, Brazil.

### Functional tests

#### Intraperitoneal glucose tolerance test (GTT)

After fasting, rats were administered an i.p. glucose injection (2 g/kg of a 20% solution of D-glucose). Blood samples were collected from the tail at 0, 10, 15, 30, 60 and 120 min after the glucose injection for the measurement of blood glucose levels. The AUC of glycemia vs. time was calculated above each individual baseline (basal glycemia) to estimate glucose tolerance.

#### Intraperitoneal insulin tolerance test (ITT)

After fasting, rats were administered an i.p. insulin injection (2 IU/kg). Blood samples were collected from the tail at 0, 5, 10, 15, 20, 25 and 30 min after the insulin injection for the measurement of serum glucose levels. The K_ITT_ was calculated using the equation: 0.693/half-life. Glucose half-life was calculated from the slope of the least-squares analysis of the blood glucose concentrations during the linear phase of decay.

#### Intraperitoneal pyruvate tolerance test (PTT)

Fasted rats were administered an i.p. injection containing sodium pyruvate solution (250 mg/ml) at a dosage of 2 g/kg. Glucose levels were determined in blood collected from the tail before (0 min) and 15, 30, 60, 90, and 120 min after the pyruvate injection. The AUC of glycemia vs. time was calculated using each individual baseline (basal glycemia) to estimate glucose production after a pyruvate load.

#### VLDL production assay

The LPL inhibitor tyloxapol (Triton WR-1339; Sigma Aldrich, St Louis, USA) was dissolved in isotonic saline 0.9% (V/V) with slight agitation. After a 60-h fast, rats were weighed and received an i.p. bolus injection of tyloxapol (500 mg/kg). Blood samples were collected form the tip of the tail at 1, 2, 3 and 6 hours after tyloxapol injection, and triglyceride was measured immediately using a diagnosis kit (Labtest, Santa Lagoa, Brazil).

#### Blood sample analysis

Glucose, TGs, cholesterol, and NEFA levels were analyzed using standard commercial kits (Labtest Diagnóstica SA, MG, Brazil, for TGs and Cholesterol; HR Series NEFA-HR from Wako Chemicals, Richmond, VA, USA, for NEFA determination). Serum insulin concentration was determined by ELISA, according to the manufacturer’s instructions (#EZRMI-13K, Millipore; MA, USA).

#### Tissue sample analysis

Liver samples were quickly removed, washed with ice-cold phosphate-buffered saline (PBS), frozen with liquid nitrogen and ground in a mortar. Powdered samples were maintained at −80 °C until used.

Glycogen content was strictly measured as previously described^40^. Lipid extraction from liver and skeletal muscle (soleus) samples was performed based on a method previously described^41^. Briefly, powdered tissue samples were homogenized in a 4-mL solution of CHCl_3_ and methanol (2:1, v/v) using a rotor-stator homogenizer. The homogenates were incubated for at least 16 h at 4 °C under gentle homogenization in a closed glass tube. After this, a 0.6% NaCl solution was added to the extracts and the mixtures were centrifuged at 2000 × g for 20 min. The organic layer was then collected and dried using an Eppendorf Vacuum Concentrator Plus (Eppendorf, Hamburg, Germany). The lipids were solubilized in 200 µL of isopropanol and quantified using standard commercial kits.

The activities of LDH (EC 1.1.1.27) and β-HAD (EC 1.1.1.35) were measured using spectrophotometric assays, following standard methods described elsewhere^42, 43^. CS (EC 4.1.3.7) activity was assayed using colorimetry at 412 nm, as previously described^44^.

### Protein extraction and immunoblotting

Fragments of liver (approximately 100 mg) were removed and processed for western blotting, as previously described^40^. The primary antibodies used were as follows: anti-AKT2 (cat. # 2962), anti-ACLY (cat. #4332) and anti-Raptor (cat. #22805) from Cell Signaling Technology (Danvers, MA, USA); anti-glucose-6-phosphatase (sc-27198) and anti-PEPCK (sc32879) from Santa Cruz Biotechnologies (Santa Cruz, CA, USA); anti-FOXO1A (ab52857) and anti-FOXO1A (phospho S256) (ab131339) from Abcam (Cambridge, UK). Anti-beta actin, (cat. # A3854; Sigma Aldrich, St Louis, USA), was used as the loading control. Secondary antibodies conjugated to horseradish peroxidase (Bio-Rad Laboratories, Hercules, CA) were used, followed by chemiluminescent detection of the bands on x-ray-sensitive films. Optical densitometry of the films was performed using the Scion Image analysis software (Scion Corp., Frederick, MD, USA). All images of the western blots are shown in Supplementary Figure S1.

Total RNA was extracted from approximately 100 mg of tissue using Qiazol reagent, and total RNA was used for both the reverse transcription with random primers for the analysis of mRNA expression and the poly(A) tailing for the analysis of miRNA expression^45^.

Primer sequences used for mRNA analysis are listed in Supplementary Table S2. Values of miRNA and mRNA expression were normalized using the geometric mean calculated from the internal control gene *rpl37a* or RNU43. Fold changes were calculated using the 2-ΔΔCT method.

#### Neutral lipids staining by Oil Red O

Liver sections were processed and analyzed as previously described^46^. Briefly, liver fragments were frozen in n-hexane with liquid nitrogen, and cryostat sections (12 μm) were mounted onto aminopropyltriethoxysilane-coated glass slides. From each block, exhaustive 12-μm serial sections were obtained (with 200 μm between sections) and randomly selected for analysis, and at least 3 sections were analyzed. Glass slides with the sections were incubated with Oil Red O for 5 min at room temperature and then rinsed with water. After rinsing, glass slides were coated with water-soluble mounting medium and coverslips. Images (four different fields of each section) were captured using a bright field Nikon Eclipse E800 microscope (Nikon, Tokyo, Japan) equipped with a digital camera (Nikon FDX-35, Nikon, Tokyo, Japan). Analysis was performed using the free software Image J (http://imagej.nih.gov/ij). Colored images were converted to grayscale and densitometry was performed on the entire captured field. The section staining and image acquisition were performed in a single experimental round and analysis was performed assuming a constant background signal for all images. Data are presented as Oil Red O optical density.

#### Prediction analysis of microRNAs that target AKT2

To identify miRNAs likely to physically bind to *akt2* mRNA, which would control *akt2* expression, we used 3 available algorithms (TargetScan, miRanda and microT-CDS) designed for predicting putative target sequences in the 3′UTR of metazoan mRNA.

### Statistical analysis

All results are presented as the mean ± S.E.M. Comparisons were performed using an unpaired Student’s t-test or a one-way ANOVA followed by a Tukey-Kramer post hoc test, when appropriate (GraphPad Prism - Graph Pad Software, Inc., San Diego, USA). P values < 0.05 indicate a significant difference.

## Electronic supplementary material

Supplementary information
