# Supplementary material for: Prolonged fasting elicits increased hepatic triglyceride accumulation in rats born to dexamethasone-treated mothers
Source: Sci Rep. 2017 Sep 4;7:10367. doi: 10.1038/s41598-017-10642-1 (PMC5583317; doi:10.1038/s41598-017-10642-1)
Supplement: Supplementary file 1 — Supplementary information [file 41598_2017_10642_MOESM1_ESM.pdf]

Supporting information - Prolonged fasting elicits increased hepatic triglyceride accumulation in rats born to dexamethasone-treated mothers

Lucas Carminatti Pantaleão[1], Gilson Murata[1], Caio Jordão Teixeira[2], Tanyara Baliani Payolla[1], Junia Carolina Santos-Silva[2], Daniella Esteves Duque-Guimaraes[1], Frhancielly S. Sodré[1], Camilo Lellis-Santos[3], Juliana Camargo Vieira[1], Dailson Nogueira de Souza[2], Patrícia Rodrigues Gomes[1], Sandra Campos Rodrigues[1], Gabriel Forato Anhe[2], Silvana Bordin[1*]

[1]Department of Physiology and Biophysics, Institute of Biomedical Sciences, University of Sao Paulo, Brazil

[2]Department of Pharmacology, Faculty of Medical Sciences, State University of Campinas

[3]Institute of Environmental, Chemical and Pharmaceutical Sciences, Federal University of São Paulo, Brazil

* sbordin@icb.usp.br

Supplementary Table S1

List of primer sets

Gene	Forward primer (5’-3’)	Reverse primer (5’-3’)
acc	TGCTTATATTGTGGATGGCTTG	TTCTACTGTCCCTTCTGGTTCC
apoB	CTGCGGTGGCAGAAATAACG	CCTTGAGCAAACCTTAGGTAGGG
cd36	TCTTCCAGCCAACGCCTTTGC	TGCACTTGCCAATGTCCAGCAC
dgat2	AAGCCCATCACCACCGTTG	TTCCTTCCAGGAGCTGGCAC
fasn	TGGTGAAGCCCAGAGGGATC	CACTTCCACACCCATGAGCG
ldha	CAGACAAGGAGCAGTGGAAGG	TAATCATGGTGGAAATGGGATG
ldhb	CCGTGTCTACAATGGTGAAGGG	TGTCCGCACTCTTCCTGAGC
mttp	TATGACCGTTTCTCCAAGAGTGG	TCAAGGTTCTCCTCTCCCTCATC
scd	TGCGTCAGCACTTTCTTACGG	GCGTGATGGTAGTTGTGGAAGC
sec22b	CGTGCTCGGAGAAATCTCGG	AACACGGCTACTGCTGCAAGC
slc2a1	GCTGTACGGCAAGATCGCTGAG	TTCAATCATGTCACCCACGCTG
srebf1	ACTGGTAGAGCACATTCCC	CAGTTGATGTAGAGGCTAAGC
YY1	CAAAGCGTTCGTTGAGAGC	TCAGGTTAGTTGACTGAGCAAAC
rpl37a	CAAGAAGGTCGGGATCGTCG	ACCAGGCAAGTCTCAGGAGGTG

Gene abbreviations and accession numbers are: acc (NM_022193), acetyl-CoA carboxylase alpha; apoB (NM_019287), apolipoprotein B; cd36 (AF072411), fatty acid translocase/CD36; dgat2 (NM_001012345), diacylglycerol O-acyltransferase 2; fasn (NM_017332), fatty acid synthase; ldha (NM_017025), lactate dehydrogenase A; ldhb (NM_012595), lactate dehydrogenase B; mttp (NM_001107727), microsomal triglyceride transfer protein; scd (NM_031841), stearoyl-coenzyme A desaturase; sec22b (NM_001025686), vesicle trafficking protein homolog B; slc2a1 (AF032120) solute carrier family 2 member 1, glut1; srebf1 (NM_001276707) sterol regulatory element binding transcription factor 1; YY1 (NM_173290), YY1 transcription factor; rpl37a (X14069), ribosomal protein L37a.

Supplementary Figure S1

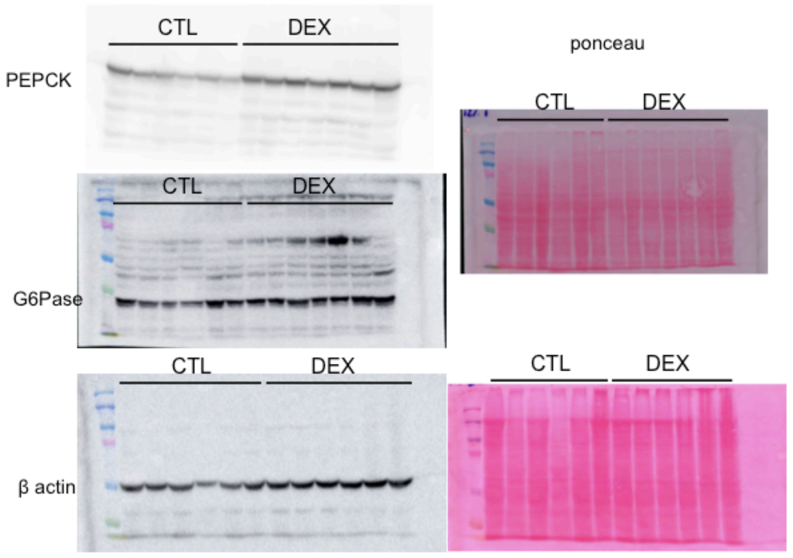

Figure 1

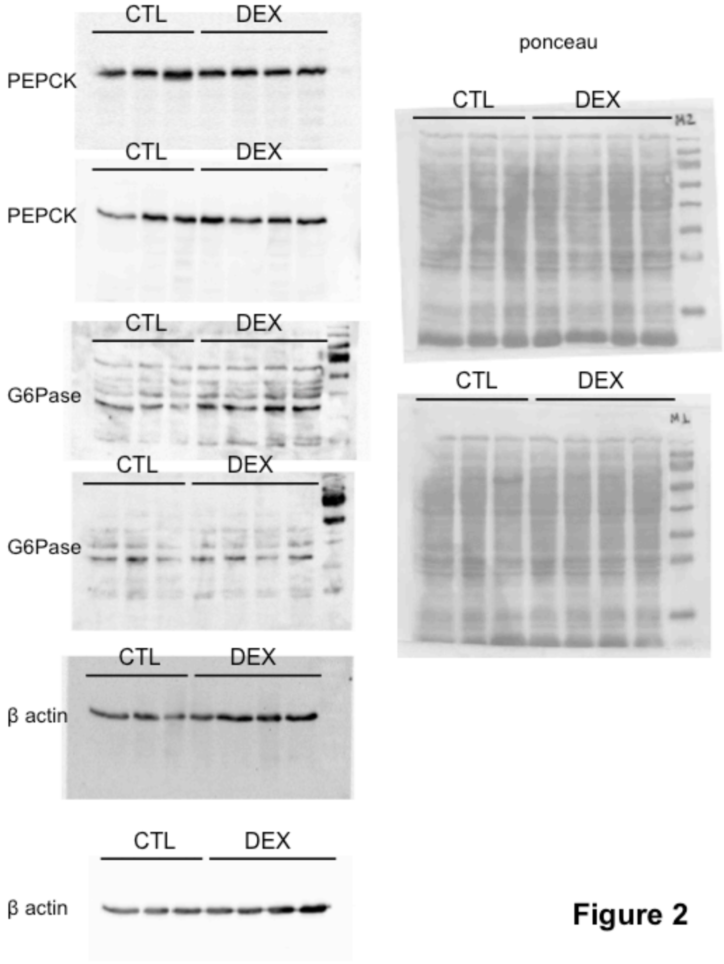

Figure 2

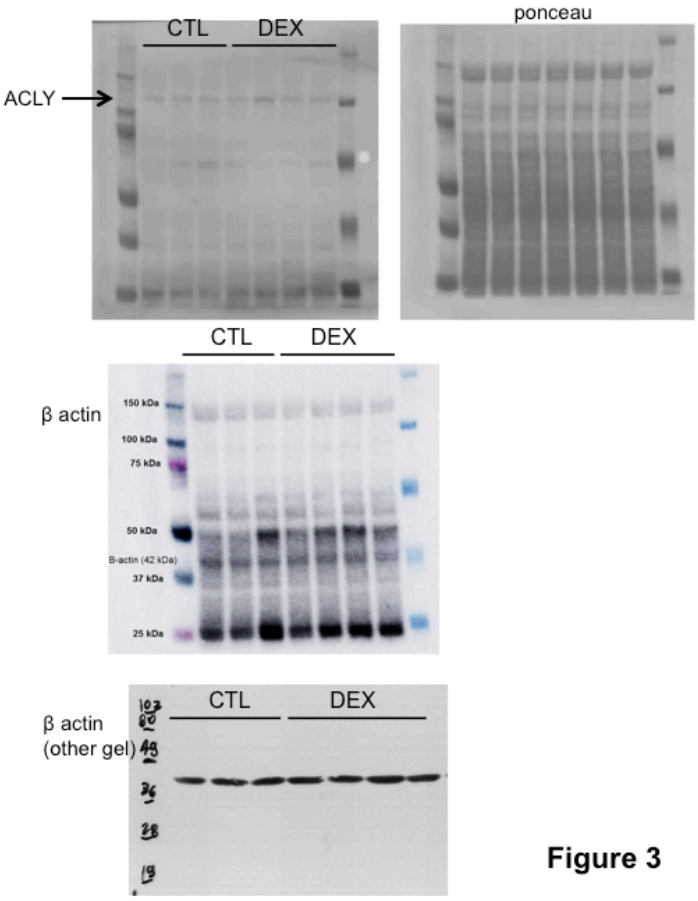
CTL
DEX
ponceau
ACLY
CTL
DEX
β actin
150 kDa
100 kDa
75 kDa
50 kDa
B-actin (42 kDa)
37 kDa
25 kDa
CTL
DEX
β actin
(other gel)

Figure 3

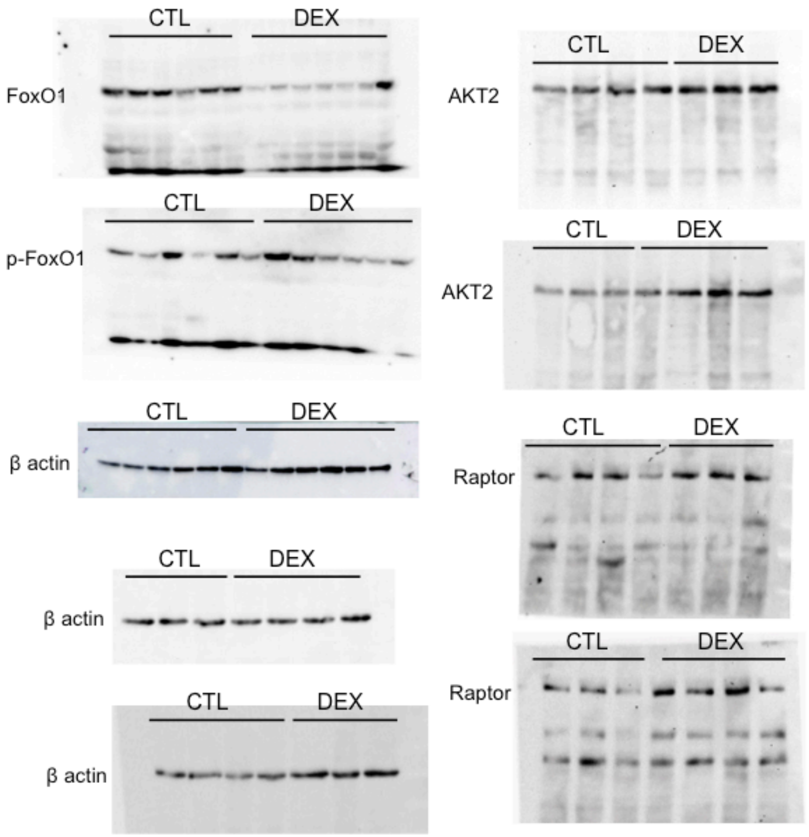
CTL
DEX
FoxO1
CTL
DEX
p-FoxO1
CTL
DEX
β actin
CTL
DEX
β actin
CTL
DEX
β actin
CTL
DEX
AKT2
CTL
DEX
AKT2
CTL
DEX
Raptor
CTL
DEX
Raptor

Figure 5

Supplementary Table S2

List of conserved miRNAs predicted by three algorithms (TargetScan, microT-CDS, and miRanda) with their putative target sequence position in 3'UTR of rat *Akt2*, type of matching between seed sequence and target sequence, and likelihood of repression calculated by TargetScan. MiRNAs in the list include transcripts shared by all three databases that are classified as conserved according to miRanda and transcripts that have conserved binding site in *Akt2* according to TargetScan. 8mer, perfect complementarity to nucleotides 2-8 of mature miRNA followed by an adenine; 7mer-m8, perfect complementarity to nucleotides 2-8 of mature miRNA; 7mer-A1, perfect complementarity to nucleotides 2-7 of mature miRNA followed by an adenine. Context++, mathematical model of miRNA targeting efficacy based on 14 unique features (Agarwal, 2015)

miRNA	Position in the UTR	seed match	context ++score
rno-miR-124-3p	570-57	8mer	-0.36
rno-miR-449a-5p	40-46	7mer-m8	-0.15
rno-miR-34a-5p	40-46	7mer-m8	-0.15
rno-miR-34c-5p	40-46,	7mer-m8	-0.14
rno-miR-150-5p	456-462	7mer-1A	-0.09
rno-miR-150-5p	228-234	7mer-m8	-0.03
rno-miR-136-5p	430-436	7mer-m8	-0.03
rno-miR-330-5p	190-196	7mer-m8	-0.02
rno-miR-326-3p	190-196	7mer-m8	-0.02
rno-miR-136-5p	236-242	7mer-1A	-0.01
rno-miR-338-3p	882-888	7mer-m8	-0.05
